# Association of Lung Fibrotic Changes and Cardiological Dysfunction with Comorbidities in Long COVID-19 Cohort

**DOI:** 10.3390/ijerph20032567

**Published:** 2023-01-31

**Authors:** Ainur T. Tauekelova, Zhanar Kalila, Akerke Bakhtiyar, Zarina Sautbayeva, Polina Len, Aliya Sailybayeva, Sadyk Khamitov, Nazira Kadroldinova, Natasha S. Barteneva, Makhabbat S. Bekbossynova

**Affiliations:** 1 National Research Center for Cardiac Surgery, Astana 010000, Kazakhstan; 2 School of Sciences and Humanities, Nazarbayev University, Astana 010000, Kazakhstan; 3 School of Medicine, Nazarbayev University, Astana 020000, Kazakhstan

**Keywords:** long COVID-19, post-COVID-19, hypertension, diabetes, lung sequelae, cardiovascular sequalae, persisting IgM against SARS-CoV-2

## Abstract

**Background**. Long COVID-19 symptoms appeared in many COVID-19 survivors. However, the prevalence and symptoms associated with long COVID-19 and its comorbidities have not been established. **Methods**. In total, 312 patients with long COVID-19 from 21 primary care centers were included in the study. At the six-month follow-up, their lung function was assessed by computerized tomography (CT) and spirometry, whereas cardiac function was assessed by elec-trocardiogram (ECG), Holter ECG, echocardiography, 24 h blood pressure monitoring, and a six-minute walk test (6MWT). **Results**. Of the 312 persons investigated, significantly higher sys-tolic and diastolic blood pressure, left ventricular hypertrophy, and elevated NT-proBNP were revealed in participants with hypertension or type 2 diabetes. Left ventricular diastolic dysfunc-tion was more frequently present in patients with hypertension. The most common registered CT abnormalities were fibrotic changes (83, 36.6%) and mediastinal lymphadenopathy (23, 10.1%). Among the tested biochemical parameters, three associations were found in long COVID-19 patients with hypertension but not diabetes: increased hemoglobin, fibrinogen, and ferritin. Nine patients had persisting IgM antibodies to SARS-CoV-2. **Conclusions**. We demon-strated a strong association between signs of cardiac dysfunction and lung fibrotic changes with comorbidities in a cohort of long COVID-19 subjects.

## 1. Introduction

The COVID-19 disease, resulting from infection with SARS-CoV-2, is highly contagious and may spread asymptomatically or presymptomatically [1,2,3]. Although it was initially reported as a cluster of respiratory tract disease and pneumonia cases, it quickly became apparent that its clinical course is often complicated by cardiovascular manifestations, including heart failure, myocardial damage, arterial and venous thrombosis, and arrhythmias [4,5,6,7]. There is a growing concern regarding the potential for COVID-19 infection to contribute to a burden of chronic cardiovascular and respiratory symptoms among recovered individuals [8,9]. Those multi-systemic symptoms can persist for months after diagnosis of acute COVID-19 infection [10,11,12]. The SARS-CoV-2 virus appears to be unique among coronaviruses due to the high transmission rate and atypical inflammatory response pattern leading in severe cases to the cytokine storm phenomenon [13]. Long COVID-19 or post-COVID-19 syndrome describes symptoms lasting for more than three months after the first COVID-19 symptoms onset [14], and there is a critical need to evaluate and understand the potential long-term implications of COVID-19 [10,15,16,17]. The most frequent symptoms of long COVID-19 are shortness of breath and fatigue, joint pain, and chest pain [18,19,20,21,22,23].

Many meta-analyses identified that the elderly population with pre-existing disease conditions such as type 2 diabetes mellitus [24,25] and hypertension [26,27] are found to be at an increased risk of getting COVID-19 infection and its complications [28]. COVID-19 has been linked to multiple extra-respiratory symptoms more prevalent in patients with positive cardiovascular disease history [29]. Long-lasting alternations in pulmonary function, including the reduced diffusing capacity of lungs and the restrictive syndrome, were observed in patients surviving COVID-19 pneumonia during a 6-month and 12-month follow-up period [30,31,32,33]. However, long COVID-19 affects even mild-to-moderate cases of survivors of COVID-19, with pulmonary sequelae reported after recovery [34]. The symptoms of long COVID-19 are still ill-defined, with patients describing fluctuating and disparate symptoms [15].

This paper reports results from an observational study of 312 patients with long COVID-19 conducted in January 2021. We aim to determine the prevalence and extent of persisting long COVID-19 symptoms over six months, emphasizing the patient’s cardiovascular and lung sequelae of COVID-19.

## 2. Materials and Methods

### 2.1. Study Design and Participants

**Figure 1:**
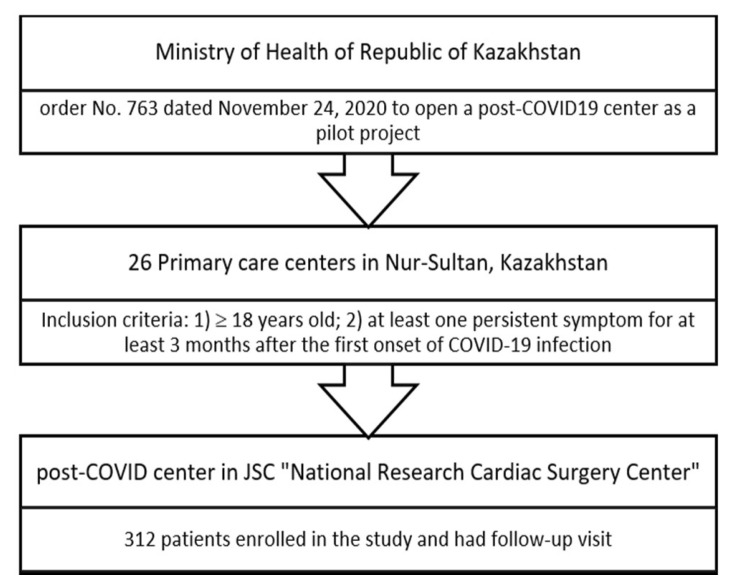
Study flow diagram.

This is an observational study that was conducted in January 2021 based on a pilot project of the post-COVID-19 center at JSC National Research Cardiac Surgery Center in Astana, Kazakhstan. Participants were selected if they had any persistent symptoms for at least three months from the first onset of the disease. The 312 selected patients were referred to the 6-month follow-up visit to JSC “National Research Cardiac Surgery Center”. The study inclusion criteria: verified diagnosis of acute COVID-19 confirmed by PCR and any persistent symptoms of long COVID-19 for at least three months from the first onset of the disease, including fatigue, shortness of breath, headache, muscle pain, brain fog, diarrhea, neurological symptoms, and skin rash (Figure 1). The exclusion criteria for the study were: (1) unconfirmed diagnosis of COVID-19 on admission; (2) negative results of PCR test for COVID-19; (3) confirmed alternative diagnosis. Participants were referred from the primary care centers of Astana (Kazakhstan) to the post-COVID-19 center.

The study was approved by the National Research Center Cardiac Surgery Ethical Committee (Protocol #01-97/2021 from 22/04/21, accessed on 22 April 2021). All participants of the study provided written informed consent. No compensation was received by participants (only reimbursement of travelling costs).

### 2.2. Clinical Assessment

All participants were given a questionnaire with items on demographic information, current complaints, comorbidities, and medications (Supplementary Data S1) and a Chalder Fatigue Scale (CFS) questionnaire (Supplementary Data S2) for completion. The confirmation of COVID-19 infection was done by RT-PCR assay on nose/throat swabs or sputum samples using CFX96 R Real-Time System (Bio-Rad Laboratories Inc., Hercules. CA, USA). Additionally, SARS-CoV-2 IgG, SARS-CoV-2 IgM, cytomegalovirus (CMV) IgG, and CMV IgM antibodies were quantified by ELISA test.

Vital signs of participants, including blood pressure and heart rate, were taken by one of three physicians participating in data collection. Hematological, clinical chemistry and biochemistry parameters were measured using Sysmex CS-2500 (Sysmex, Japan) and COBAS 6000 (Roche, Switzerland). The hematological analysis included complete blood count, hemoglobin, glycohemoglobin A_1c_ (HbA_1c_), and C-reactive protein (CRP); for biochemistry panel serum creatinine, blood urea, total cholesterol, glucose, vitamins B12 and 25(OH)D, and natriuretic (NT)-proBNP peptide, and alanine transaminase (ALT) were included; for coagulation profile—fibrinogen, D-Dimer (DD), and ferritin.

To all participants, computerized chest tomography (CT) was proposed, but 85 participants (28.3%) refused due to personal reasons. CT scanning was performed on a Somatom Definition AS instrument (Siemens Healthcare, Germany); the voltage was 80 to 120 kV, and the slice thickness was 1 mm. The standard supine patient position was used. A workstation with syngo.via software was applied to analyze CT images. Intravenous contrast bolus injection was performed using an automatic bolus-free CT injector (Ohio Tandem^TM^ (Ulrich Medical, Ulm, Germany; speed 4–5 mL/s), followed by the introduction of saline solution (50 mL). The dose of the contrast agent was calculated based on the patient’s weight. All CT images were reviewed in random order by one senior cardiothoracic radiologist.

Cardiac function was assessed by electrocardiogram (ECG), Holter ECG, echocardiography, and 24 h blood pressure monitoring in all participants. A six-minute walk test (6MWT) was conducted on 308 participants at the follow-up visit. The medical records of each participant were reviewed independently by three physicians (including a cardiologist with more than ten years of experience) of the hospital.

### 2.3. Statistical Analysis

Statistical analysis was performed in parallel using R vs. 4.1.2 (2021-11-01) and GraphPad Prism vs. 8.4.3 (Dotmatics, USA). Normality was assessed using the Shapiro test. Two-group comparison of continuous variables was performed using the T-test for normally distributed data and the Mann–Whitney–Wilcoxon test if the condition of normality was violated. For multiple comparisons, we applied ANOVA and Kruskal–Wallis tests with Tukey and Dunn tests as post hoc methods. Hochberg *p*-value adjustment was used to reduce the false discovery rate during multiple comparisons. Categorical variables were analyzed with Fisher’s Exact test. *p*-values less than 0.05 were considered significant.

## 3. Results

### 3.1. Cohort Description

A total of 312 participants who recovered from COVID-19 infection were included in the study. The mean time for the post-COVID-19 functional and laboratory test assessments was 5.8 ± 0.9 months from the onset of the first symptoms of COVID-19 infection. In total, 111 participants were hospitalized during the active COVID-19 infection, while 201 participants were treated at home. Clinical characteristics and comorbidities are reported in Table 1 from the most prevalent to the least.

### 3.2. Clinical Manifestations at the Follow-Up

Clinical manifestations of participants at the follow-up are reported in Table 2. The symptoms are presented from the most common to the least. Overall, there was no significant difference between post-COVID-19 clinical manifestations among female and male participants, except for memory dysfunction and anxiety.

Among the tested parameters, those positively correlated with comorbid conditions include age, body mass index (BMI), and inflammatory C-reactive protein (CRP) [35], neutrophils, which were shown to be elevated in severe COVID-19 patients [36], and levels of glucose (fasting and HbA1c). We also checked the level of *N*-terminal pro-B-type natriuretic peptide (NT-proBNP), which is frequently elevated during acute COVID-19 [37], and found a positive association with hypertension in long COVID-19. A wealth of evidence also points out that the hyperglycemic status (fasting glucose and Hb1c levels) makes individuals more sensitive to infections and COVID-19 [38,39].

**Figure 2:**
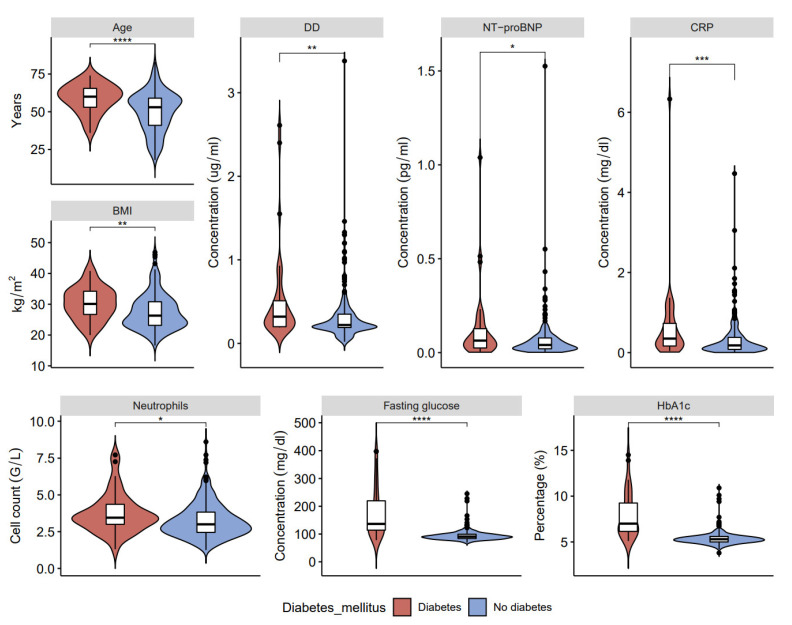
Clinical characteristics and laboratory results of blood samples in post-COVID-19 patients with and without type 2 diabetes: age, D-dimers (DD), ventricular natriuretic peptide (NT-proBNP), C-reactive protein (CRP), body mass index (BMI), neutrophils, fasting glucose, and glycated hemoglobin (HbA1c) (* *p* < 0.05, ** *p* < 0.01, *** *p* < 0.001, **** *p* < 0.0001).

**Figure 3:**
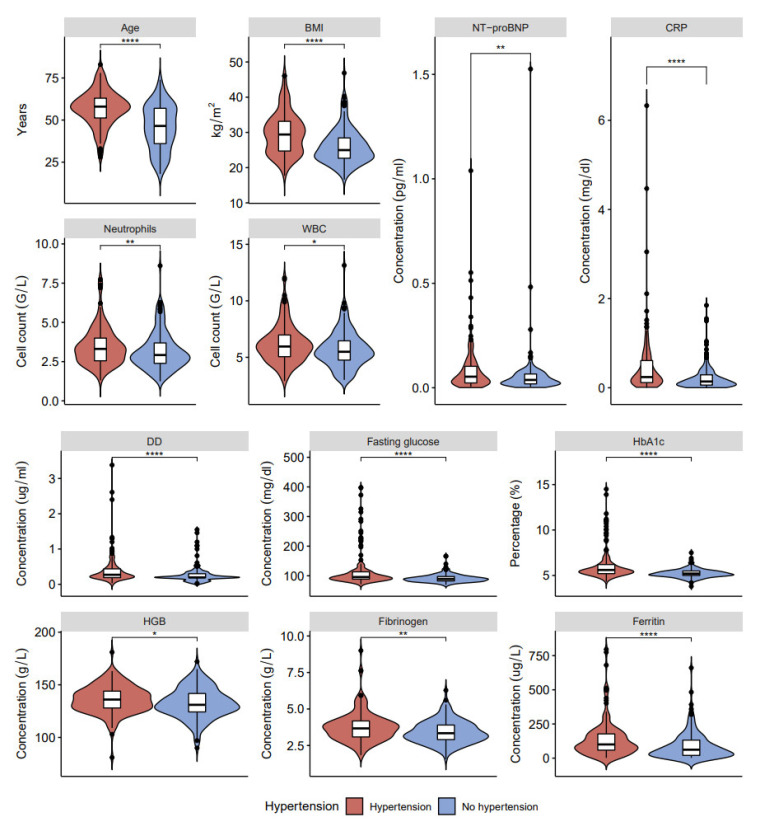
Clinical characteristics and laboratory results of blood samples in post-COVID-19 patients with and without hypertension: age, BMI, NT-proBNP, CRP, neutrophils, white blood cells (WBC), DD, fasting glucose, HbA1c, HGB, fibrinogen, and ferritin (* *p* < 0.05, ** *p* < 0.01, **** *p* < 0.0001).

**Figure 4:**
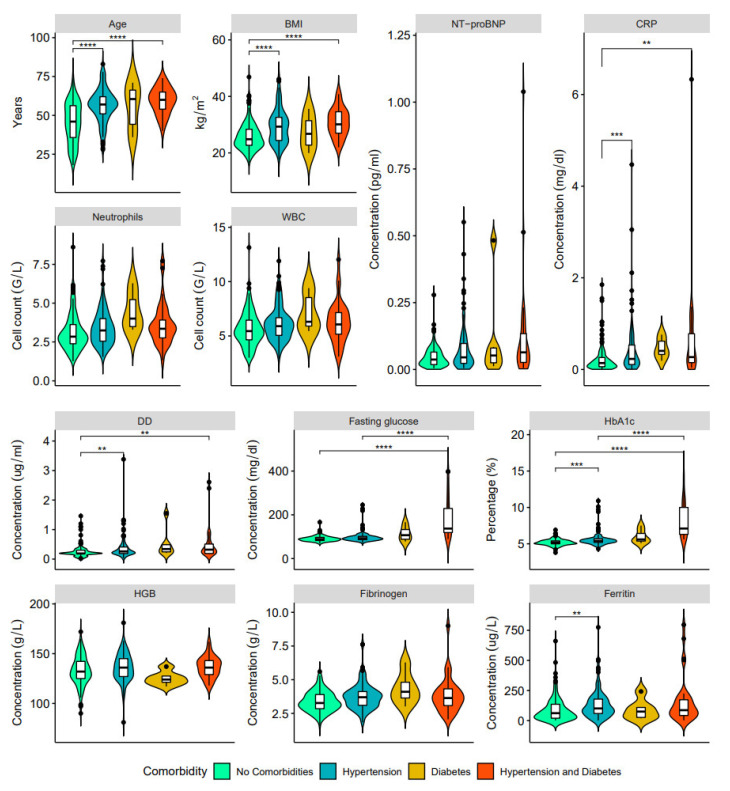
Clinical characteristics and laboratory results of blood samples in post-COVD-19 patients with no comorbidities (green), hypertension only (blue), type 2 diabetes mellitus only (orange) and both hypertension and type 2 diabetes (red): age, BMI, NT-proBNP, CRP, neutrophils, WBC, DD, fasting glucose, HbA1c, HGB, fibrinogen, and ferritin (** *p* < 0.01, *** *p* < 0.001, **** *p* < 0.0001).

In our study, patients with long COVID-19 and hypertension show three associations that were not found in patients with long COVID-19 and diabetes: increased hemoglobin, fibrinogen, and ferritin (Figure 2, Figure 3 and Figure 4, data in Supplementary Tables S1 and S2). On the other hand, higher levels of fasting glucose, HbA1c, and CRP are attributed to having both comorbid states rather than one.

Immunological test results showed that SARS-CoV19 IgM antibodies were present in nine subjects (2.88%). Among those, five participants had follow-ups in the 5th month after the onset of the disease, while the other four participants had follow-ups in the 6th month. SARS-CoV19 IgG antibodies were present in 241 subjects (77.2%) and were <10 AH/mL in 71 participants (22.8%). Meanwhile, increased CMV IgG titers defined as >0.5 U/mL were present in almost all 312 participants.

### 3.3. Lung Function Screening

**Figure 5:**
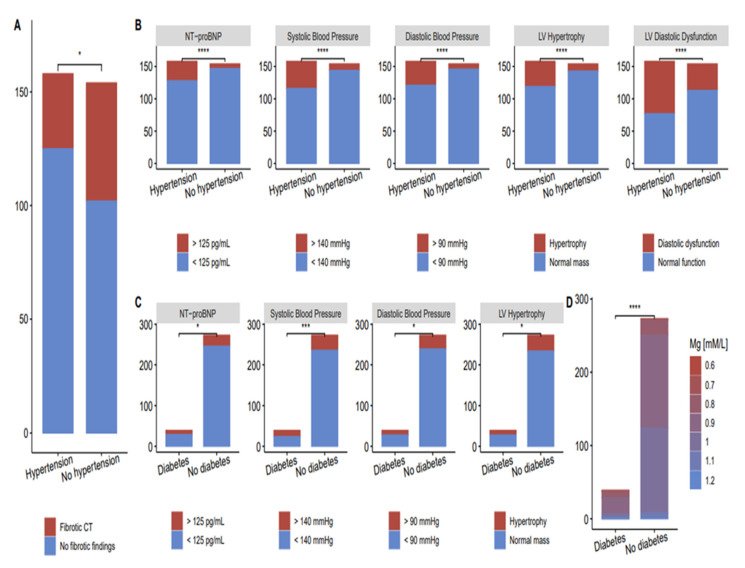
Fibrotic changes and cardiac abnormalities in patients with and without comorbidities. (**A)** Fibrotic changes in CT scan of post-COVID-19 patients with and without hypertension; (**B**) Cardiac abnormalities in post-COVID-19 patients with and without hypertension; (**C**) Cardiac abnormalities in post-COVID-19 patients with and without type 2 diabetes; (**D**) Fibrotic changes in CT scan of post-COVID-19 patients with and without type 2 diabetes (* *p* < 0.05, *** *p* < 0.001, **** *p* < 0.0001).

**Chest CT.** At the follow-up visit, with a mean of 5.8 ± 0.9 months, a chest CT was conducted on 227 subjects (72.8%). Twelve subjects (5.29%) had normal chest CTs. The most common registered CT abnormalities were fibrotic changes (83, 36.6%) and mediastinal lymphadenopathy (23, 10.1%). Most of the participants had more than one CT change. The median age of participants with lung abnormalities in chest CT was 55 years (IQR, 7). Other CT abnormalities included polysegmental pneumonia, bronchiectasis, emphysema, bullous changes, atelectasis, and interstitial pneumonia. These chest CT findings do not have a comorbidity-associated predisposition. However, fibrotic changes were found in 55 out of 158 patients with hypertension, compared to 30 out of 154 participants without hypertension (Figure 5).

One hundred and seven participants had chest CT during active COVID-19 infection, with the mean percentage of lung defects at 36.5 ± 22.6%. Among those participants, 87 subjects (39.6%) underwent chest CT at the follow-up, and 84 (96.6%) subjects had residual lung abnormalities detected at the follow-up.

**Figure 6:**
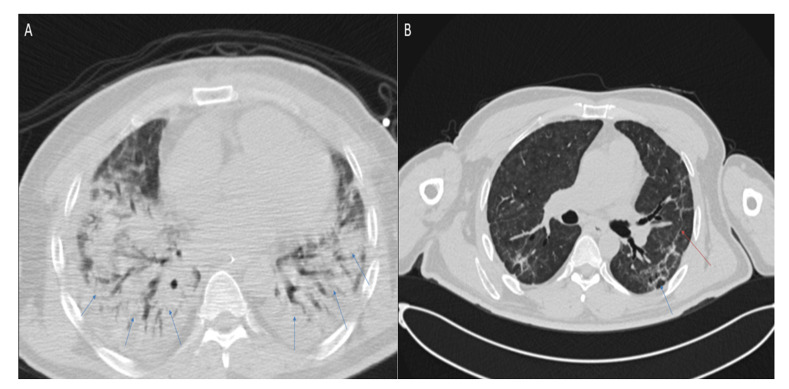
Axial chest CT images (lung window) of a 47-year-old patient obtained (**A**) at hospital admission with confirmed acute COVID-19 shows signs of bilateral polysegmental pneumonia and ground-glass opacities. Lung damage ~80%. Blue arrows indicate infiltration of the lung parenchyma; (**B**) six months after acute COVID-19 shows traction wall thickening of segmental and subsegmental bronchi in the parenchyma of both lungs. Fibrous cords, irregularly linear forms, without signs of infiltration, mediastinal lymphadenopathy. Fibrosis is marked with a red arrow, dilated bronchus with a blue arrow.

The example of lung sequelae revealed by the CT scan is provided in Figure 6. During acute COVID-19, the patient experienced a fever, general weakness, and malaise. Subsequently, he noted a deterioration in general well-being, the addition of shortness of breath with minimal physical exertion, and the fever persisted. Taking into account the increase in respiratory failure, he was urgently hospitalized. A CT scan of the chest revealed a picture of bilateral polysegmental pneumonia in the consolidation stage (viral etiology—COVID-19), with the degree of lung damage at 80%.

**Spirometry test.** At the follow-up visit, all participants had a spirometry test, and 143 subjects (45.8%) had spirometry without abnormalities. Out of 169 subjects (54.2%) with abnormal spirometry, 118 subjects (37.8%) had a restrictive pattern and 43 subjects (13.8%) had mixed results with a mostly restrictive pattern. Only eight subjects (2.6%) had obstructive defects detected in spirometry. Regarding participants with respiratory comorbidities (nine), there was one participant with restrictive, one participant with obstructive, and four participants with mixed and mostly restrictive defects. A total of 67 participants (60.3%) out of 111 hospitalized participants had abnormal spirometry results.

**Correlation between chest radiographs and 6MWT with lung function.** In total, 125 (58.1%) out of 215 subjects with abnormal chest CT at the follow-up had abnormalities in the spirometry test. Eighty-nine subjects (71.2%) had a restrictive pattern, thirty subjects (24%) had mixed with a mostly restrictive pattern, while six subjects (4.8%) had an obstructive pattern in spirometry.

The median 6MWT in participants with abnormal spirometry was 381 (310;416) meters, which was significantly different from participants with normal spirometry (400 (350;450) meters, *p* = 0.004). Fourteen (77.8%) out of eighteen subjects with specific respiratory symptoms, including wheezing, dry cough, and cough with sputum, had abnormalities in chest CT at the follow-up. Meanwhile, 32 (68.9%) out of 45 subjects with dyspnea on exertion and 15 (83.3%) out of 18 subjects with dyspnea on rest had an abnormal chest CT scan.

### 3.4. Cardiac Screening

All 312 participants underwent a cardiac screening (Table 3); 24 h blood pressure monitoring revealed significantly higher systolic and diastolic blood pressure and left ventricular hypertrophy. Cardiac abnormalities in patients with hypertension and type 2 diabetes compared to participants with no comorbidities are shown in Figure 5B,C, respectively. Left ventricular diastolic dysfunction is more frequently present in patients with hypertension.

## 4. Discussion

In our study, six months after acute illness with COVID-19, most of the patients still meet the criteria for long COVID-19 and show at least one persistent symptom. These results are mainly in line with studies reporting the prevalence of long COVID-19 in patients 3 to 6 months after COVID-19 [40,41,42,43]. Long COVID-19 symptoms have similarities with chronic fatigue syndrome developed after viral infections [44,45] and may afflict a wide range of organs, with the most common symptoms being chronic fatigue, shortness of breath, and cognitive impairments [20,21,22,23].

The pathophysiology of long COVID-19 respiratory and multi-organ sequelae has a fundamental knowledge gap that must be addressed [46]. Viral infections are considered to be cofactors for the initiation and exacerbation of lung fibrosis [47], and clinical evidence suggesting cardiovascular involvement has been reported for essentially all known viral infections [48]. Observational studies revealed a high percentage of long COVID-19 sequelae, such as respiratory problems, lung fibrosis [49,50], and cardiovascular problems [51]. Moreover, long COVID-19 affects even mild-to-moderate cases of survivors of COVID-19, with pulmonary sequelae reported after recovery [34]. Furthermore, our study demonstrated that deranged blood indices (increased neutrophils, WBC, changed coagulation profile-significantly increased DD; fasting glucose, HbA1c, HGB) and raised inflammatory marker (CRP) are associated with pre-existing comorbidities, specifically, hypertension and diabetes. Thus, HbA1c is considered the gold standard for evaluating blood glucose levels [52] associated with inflammation, hypercoagulability, blood oxygen saturation, and higher mortality rate in COVID-19 patients [34].

Hypertension affects more than a quarter of the global population [53,54] and has been identified as a major risk factor for COVID-19 severity [55,56] and a higher risk of dying from COVID-19 [57]. Moreover, hypertension is closely associated with other comorbidities, predominantly obesity and diabetes, and pulmonary hypertension may induce additional myocardial damage [58]. In this study, besides the above-mentioned significant changes in blood and inflammatory markers that coincided with other comorbidities (diabetes), hypertension also revealed significant differences in fibrinogen (Fbg) and ferritin levels. Changes in Fbg and ferritin levels can be associated with cell stress and damage and substantial rearrangement in blood vessel walls [59,60,61]. The abnormal Fbg levels strongly correlate with COVID-19 disease severity [62], and assessment of Fbg levels at admission is essential for determining the prognosis of COVID-19 patients [63]. Abnormal Fbg levels are observed in post-COVID-19 cohorts by different groups [64]. Cardiovascular changes associated with COVID-19 may lead to left ventricular dysfunction [65], which in our cohort had a higher prevalence in convalescent patients with hypertension.

Cardiac screening showed a mean ejection fraction of 61 ± 4.55%, less than in participants observed 1–4 weeks after discharge [66]. This can be explained by a higher prevalence of chronic heart diseases in our cohort of patients, including hypertension and CAD. Interestingly, our study revealed new cases of left ventricular hypertrophy and diastolic dysfunction of LV in participants with no pre-existing cardiovascular disorders. In addition, 19 participants had newly diagnosed hypertension during the follow-up. The patients with newly diagnosed hypertension were young (median, 45 years) and had no pre-existing cardiovascular comorbidities before the follow-up. However, echocardiography revealed long-term complications in some patients, meaning that hypertension might have been before the 6-month follow-up and COVID-19 infection. In addition to hypertension and LV dysfunction, our study found nine new cases of rhythm disturbances detected in 24 h BP monitoring. Newly detected rhythm disturbances included atrial fibrillation (AF), AB block II degree, sinoatrial (SA) block, and RBBB.

Although our cohort was limited to establishing the time of the onset of hypertension in the post-COVID-19 period, our findings in patients with no pre-existing cardiac comorbidities and standard echocardiography suggest the presence of the long-term effect of COVID-19 on cardiac function. The evidence of cardiac injury in post-COVID-19 patients (left ventricular ejection fraction and diastolic dysfunction) and the appearance of arrhythmias corroborate the findings of other investigators [67,68]. The arrhythmias in post-COVID-19 patients were found to be associated with COVID-19 disease severity [69].

In patients with COVID-19, the viral infection may initiate inflammation with subsequent fibrosis, and cardiac inflammation and fibrosis are major pathological mechanisms leading to heart failure [70]. Non-invasive imaging, blood analysis for fibrosis markers, and endomyocardial biopsy (EMB) are used to detect fibrosis [71]. The primary imaging technique in contemporary heart failure management is echocardiography; however, it provides little information about the extent of fibrosis [72]. To evaluate the cardiac and lung changes, we used echocardiography and chest CT. The question of whether the lung is a target organ for diabetes mellitus has been intensively discussed, and a body of clinical and experimental evidence indicates that fibrosis and other pulmonary complications are more prevalent than were early recognized [73,74,75]. Critically fibrotic changes on follow-up CT scans were significantly associated with hypertension but not diabetes (Figure 5). The presence of diabetes and new-onset diabetes was an independent factor associated with poor outcomes during different coronavirus infections—SARS-CoV-1 [76], MERS-CoV [77], and SARS-CoV-2 [24]. In our study, hypertension had a significant association with fibrotic changes on CT-scan, whereas diabetes type II did not. One of the possible explanations can be a small percentage of patients with type II diabetes in our long COVID-19 cohort. However, we may also speculate that the fibrotic changes in post-COVID-19 develop faster when blood vessels are already affected by hypertension. It was reported that abnormal lung CT findings could be present even in asymptomatic or mild COVID-19 patients [78]. The association of radiological CT findings with hypertension in long COVID-19 makes hypertension, combined with other results (high fibrinogen, DD, etc.), a possible early marker of developing fibrotic lung changes.

Laboratory tests identified the long-term persistence of IgM antibodies in 9 patients from our cohort (5–6 months). The IgM antibodies at COVID-19 infection peak early and rapidly decline [79,80,81]. IgM seropositivity was found to be correlating with symptom duration [78]. Moreover, the long-term persistence of anti-SARS-CoV-2 IgM antibodies was recently reported by Bichara and colleagues [82], who observed an 8-month-long persistence of the IgM antibodies after COVID-19 infection in two patients. The prolonged persistence of anti-viral IgM antibodies was described after different acute viral infections and live viral vaccines: the Japanese encephalitis live vaccine [83], acute hepatitis A infection [84], dengue virus infection [85], Zika virus > two years [86], yellow fever virus vaccine > 3–4 years [87], and in cerebrospinal liquid and blood of patients after West Nile virus infection [88,89,90,91] in some cases up to 8 years after infection. The finding of the long persistence of IgM antibodies in some participants of the post-COVID-19 cohort warrants additional investigation, particularly the determination of whether the persistence of IgM is related to persistent infection with SARS-CoV-2. However, the finding of elevated IgG anti-CMV antibodies in most of the cohort should be interpreted with caution due to the possibility of cross-reactivity between viral epitopes and human tissue antigens [92,93].

The main strength of our observational cohort study was the inclusion of consecutive patients with laboratory-confirmed COVID-19, and multi-organ post-COVID-19 manifestations were evaluated, particularly cardiological and lung changes.

**Study Limitations** include: (1) a single study location and the observational nature of the study; patients only with persistent symptoms were selected for the study, and there was no control group for comparison of patients with and without the long COVID-19 syndrome; (2) the absence of baseline health data and CT-imaging data before the infection limits the assessment of changes in patient’s results; (3) social information of patients, including the number of years of smoking, alcohol use, and lifestyle, was not reported, which might have a confounding effect on some of the results. Moreover, we did not include individuals with a mild or asymptomatic phenotype of the disease; (4) the underrepresentation of some ethnic groups within our cohort (prevalence of Asian ethnic group) may limit the translation of our findings to populations with different ethnic structures.

## 5. Conclusions

The data in this study permit a 6-month assessment of long COVID-19 sequelae. Long-term prospective studies of longer duration are still needed. With the continuing outbreaks of SARS-CoV-2 infection in many countries, it is expected that the patients’ burden of having long COVID-19 respiratory and cardiovascular sequelae is going to increase and lead to another public health crisis following the current pandemic. Thus, further research is critical for evaluating the long-term consequences of COVID-19. The preliminary study presented here can be prolonged to 12 and 24 follow-ups. This will enable us to better understand the long-term effects of long COVID-19 and identify symptoms significant for medical treatment and observation.

## Figures and Tables

**Table 1:** Clinical characteristics and comorbidities in participants after COVID-19 infection.

	Total (*n* = 312)	Female (*n* = 211)	Male (*n* = 101) *p*-Value
Age †, years	54 (42.5;60)	54 (44;60)	55 (40;61) ns
BMI †, kg/m^2^	26.9 (23.4;31.1)	26.9 (23.1;31.6)	26.7 (24;30.4) ns
Obesity class			
Overweight			
(BMI 25.0–29.9)	90 (28.9)	57 (27.0)	33 (32.7) ns
Obese			
(BMI 30.0)	103 (33.0)	73 (34.6)	30 (29.7) ns
CFS ≥ 4	243 (77.9)	173 (82.0)	70 (69.31) 0.013
Hypertension	158 (50.6)	112 (53.1)	61 (60.4) ns
GI disorders	74 (23.7)	49 (23.2)	25 (24.7) ns
Neurological disorders	43 (13.8)	31 (14.7)	12 (11.9) ns
Type 2 Diabetes	40 (12.8)	27 (12.8)	12 (12.9) ns
CAD	30 (9.62)	15 (7.10)	15 (14.9) 0.039
Endocrine disorders	17 (5.45)	16 (7.58)	1 (0.99) 0.015
Heart Failure	14 (4.49)	8 (3.79)	6 (5.94) ns
UTI disorders	14 (4.49)	7 (3.32)	7 (6.93) ns
Rhythm disturbances	6 (1.92)	3 (1.42)	3 (1.42) ns
Asthma	7 (2.24)	6 (2.84)	1 (0.99) ns
COPD	2 (0.64)	1 (0.47)	1 (0.99) ns

Note: Except where indicated, data are numbers of participants, with percentages in parentheses. † Data are medians, with interquartile ranges in parentheses. *p*-values comparing female and male participants were determined with the Fisher’s Exact test or independent-samples *t* test, or Mann–Whitney *U* test; ns – not significant. CAD—coronary artery disease; CFS—Chalder Fatigue Score; UTI—urinary tract infection; Rhythm disturbances—sinus tachycardia, atrial tachycardia, ventricular extrasystoles, atrial fibrillation, atrioventricular blockade; COPD—Chronic Obstructive Pulmonary Disease; GI disorders included chronic cholecystitis, pancreatitis, steatohepatitis, and atrophic gastritis; neurological disorders included encephalopathy of mixed etiology; endocrine disorders included hypothyroidism.

**Table 2:** Participant-reported clinical manifestations persistent after COVID-19 infection at median of 6 months follow-up.

	Total (*n* = 312)	Female (*n* = 211)	Male (*n* = 101)	*p*-Value
Fatigue	220 (70.5)	143 (67.8)	77 (76.2)	ns
Tiredness	180 (57.7)	118 (55.9)	62 (61.5)	ns
Sleep disturbances	168 (53.9)	116 (55.0)	52 (51.5)	ns
Muscle pain	109 (34.9)	78 (37.0)	31 (30.7)	ns
Memory dysfunction	108 (34.6)	81 (38.4)	27 (26.7)	0.04
Dizziness	107 (34.3)	80 (37.9)	27 (26.7)	0.05
Headache	79 (25.3)	59 (28.0)	20 (19.8)	ns
BP instability	62 (19.9)	38 (18.0)	24 (23.8)	ns
Palpitation	47 (15.1)	34 (16.1)	13 (12.9)	ns
Dyspnea on exertion	45 (14.4)	31 (14.7)	14 (13.9)	ns
Joint pain	39 (12.5)	30 (14.2)	9 (8.9)	ns
Increased sweating	39 (12.5)	29 (13.7)	10 (9.9)	ns
Anxiety	28 (8.97)	26 (12.32)	2 (1.98)	0.003
Hair loss	27 (8.65)	19 (9.00)	8 (7.92)	ns
Dyspnea on rest	18 (5.77)	11 (5.21)	7 (6.93)	ns
Decreased vision	18 (5.77)	12 (5.69)	6 (5.94)	ns
GI symptoms	16 (5.13)	12 (5.69)	4 (3.96)	ns
Chest pain in rest	12 (3.85)	11 (5.21)	1 (0.99)	ns
Daytime sleeping	11 (3.53)	6 (2.84)	5 (4.95)	ns
Cough with sputum	9 (2.88)	7 (3.32)	2 (1.98)	ns
Mood change	8 (2.56)	5 (2.37)	3 (2.97)	ns
Chest pain on exertion	7 (2.24)	5 (2.37)	2 (1.98)	ns
Dry cough	7 (2.24)	7 (3.32)	0	ns
Numbness in extremity	7 (2.24)	5 (2.37)	2 (1.98)	ns
Decreased hearing	6 (1.92)	6 (2.84)	0	ns
Tremor in extremity	5 (1.60)	3 (1.42)	2 (1.98)	ns
Impaired sense of smell	5 (1.60)	3 (1.42)	2 (1.98)	ns
Increased weight	4 (1.28)	3 (1.42)	1 (0.99)	ns
Decreased concentration	3 (0.96)	2 (0.95)	1 (0.99)	ns
Wheezing	2 (0.67)	1 (0.47)	1 (0.99)	ns
Nervousness	2 (0.64)	2 (0.95)	0	ns
Presyncope	2 (0.64)	2 (0.95)	0	ns
Syncope	2 (0.64)	2 (0.95)	0	ns
Impaired sense of taste	2 (0.64)	1 (0.47)	1 (0.99)	ns
Fever	2 (0.64)	1 (0.47)	1 (0.99)	ns
Edema of extremities	2 (0.64)	2 (0.95)	0	ns

Note. Data are numbers of participants, with percentages in parentheses. *p* values comparing female and male participants were determined with the with the Fisher’s Exact test or independent-samples *t* test, or Mann–Whitney *U* test; ns – not significant. BP—blood pressure.

**Table 3:** Cardiac abnormalities detected in cardiac assessment of post-COVID-19 patients.

Serum Cardiac Biomarker	Total	
NT-proBNP > 125 pg/mL	37 (11.9)	
Electrocardiography		
Sinus bradycardia < 60 bpm	26 (8.33)	
Sinus bradycardia < 50 bpm	4 (1.28)	
	Total	Newly detected
Echocardiography		
Left ventricular ejection fraction †, %	61 ± 4.55	
Left ventricular ejection fraction <50%	5 (1.60)	
Left ventricular hypertrophy	50 (15.5)	7 (2.24)
Diastolic dysfunction of left ventricle	122 (39.1)	2 (0.64)
Dilation of right atrium	2 (0.64)	1 (0.32)
Dilation of left atrium	12 (3.85)	0
Holter electrocardiography		
Atrial fibrillation	5 (1.60)	4 (1.28)
AV block II degree	4 (0.32)	3 (0.96)
Sinoatrial block	1 (0.32)	1 (0.32)
RBBB	1 (0.32)	1 (0.32)
24 hr BPM		
Mean SBP ≥140 mmHg	71 (22.8)	19 (6.10)
Mean DBP ≥90 mm Hg	49 (15.7)	19 (6.10)

Note. Except where indicated, data are numbers of participants, with percentages in parentheses. † data are means ± standard deviation; 24 hr BPM—24 h blood pressure monitoring; AV—atrioventricular; RBBB—right bundle branch block; BPM—beats per minute; SBP—systolic blood pressure; DBP—diastolic blood pressure.

## Data Availability

The raw data supporting the conclusions of this article will be made available by the authors, without undue reservation.

## Supplementary Materials

The following supporting information can be downloaded at: https://www.mdpi.com/article/10.3390/ijerph20032567/s1, Data S1: Questionnaire with items on demographic information, current complaints, comorbidities, and medications; Data S2: Questionnaire of a Chalder Fatigue Scale (CFS) questionnaire.; Table S1: Participants-reported clinical manifestations persistent after COVID-19 infection at median of 6 months follow-up. Table S2: Comparison of clinical manifestations amongst participants with and without hypertension.
