# Supplementary material for: Association of Lung Fibrotic Changes and Cardiological Dysfunction with Comorbidities in Long COVID-19 Cohort
Source: Int J Environ Res Public Health. 2023 Jan 31;20(3):2567. doi: 10.3390/ijerph20032567 (PMC9915134; doi:10.3390/ijerph20032567)
Supplement: Supplementary file 1 [file ijerph-20-02567-s001.zip › Suppl_Data_S1_Tauekelova_et_al.pdf]

Supplemental Data S1

Questionnaire for participants

1. Surname, Name____
2. Telephone number____
3. Age ____
4. Height/weight ____
5. Allergy (indicate if any) ____

6. Indicate if you have any of those complaints

◻ Fatigue ◻ Increased sweetness ◻ Dizziness
◻ Increased/decreased blood pressure ◻ Palpitation
◻ Pain in muscle ◻ Syncope
◻ Pain in epigastric region ◻ Sleep disturbances
◻ Other (indicate)____

7. Chronic diseases (yes/no, if yes, indicate which)
   (eg. Diabetes mellitus, hypertension, asthma, gastritis, etc.)
   ____
   ____

8. Please indicate the period of coronavirus infection (day, month, duration in days)
   ____
   ____

9. Were you hospitalized during the infection period (yes/no)?
   ____
   ____
   ____

10. Did you have chest Xray or chest CT during the infection period

    ◻ Yes ◻ No

11. Please indicate any medications you take currently (if any)?
    ____
    ____
    ____
    ____
    ____

Note. Data was collected from 312 patients with post-COVID-19
