# Supplementary material for: Association of Lung Fibrotic Changes and Cardiological Dysfunction with Comorbidities in Long COVID-19 Cohort
Source: Int J Environ Res Public Health. 2023 Jan 31;20(3):2567. doi: 10.3390/ijerph20032567 (PMC9915134; doi:10.3390/ijerph20032567)
Supplement: Supplementary file 1 [file ijerph-20-02567-s001.zip › Suppl_Data_S2_Tauekelova_et_al.pdf]

Supplemental Data S2

Chalder Fatigue Scale (CFS)

Name: ______________________________________ Date: __________________

Please answer ALL the questions by ticking the answer which applies to you most closely. If you have been feeling tired for a long while, then compare yourself to how you felt when you were last well. Please tick only one box per line.

	less than usual	no more than usual	more than usual	much more than usual
Do you have problems with tiredness?				
Do you need to rest more?				
Do you feel sleepy or drowsy?				
Do you have problems starting things?				
Do you lack energy?				
Do you have less strength in your muscles?				
Do you feel weak?				
Do you have difficulties concentrating?				
Do you make slips of the tongue when speaking?				
Do you find it more difficult to find the right word?				
	better than usual	no worse than usual	worse than usual	much worse than usual
How is your memory?				

Note. Data was collected from 312 patients with post-COVID-19
