# Supplementary material for: Association of Lung Fibrotic Changes and Cardiological Dysfunction with Comorbidities in Long COVID-19 Cohort
Source: Int J Environ Res Public Health. 2023 Jan 31;20(3):2567. doi: 10.3390/ijerph20032567 (PMC9915134; doi:10.3390/ijerph20032567)
Supplement: Supplementary file 1 [file ijerph-20-02567-s001.zip › Supplemental_Tables1-2_Tauekelova_et_al.pdf]

International Journal of Environmental Research and Public Health

MDPI

Supplemental Table S1. *Comparison of clinical manifestations amongst participants with and without diabetes*

	**Total (n=312)**	**Diabetes (n=39)**	**No Diabetes (n=273)**	**p-value**
Age, years	54 (42.5;60)	60 (53;65.5)	53 (41;59)	< 0.001
BMI, kg/m^2	26.9 (23.4;31.1)	30.1 (26.7;34.2)	26.3 (23.1;30.8)	0.001
DD	0.23 (0.19;0.36)	0.32 (0.2;0.51)	0.22 (0.19;0.35)	0.001
NT-proBNP	0.043 (0.021;0.082)	0.064 (0.0247;0.127)	0.041 (0.021;0.078)	0.02
CRP	0.19 (0.09;0.41)	0.35 (0.165;0.73)	0.18 (0.08;0.38)	<0.001
Neutrophils	3.105 (2.49;3.895)	3.45 (3;4.36)	3 (2.45;3.83)	0.01
Fasting glucose	92.68 (85.04;103.07)	136 (114;220)	90.8 (84;99.3)	<0.001
HbA1c	5.3 (5.075;5.7)	7 (6.15;9.25)	5.3 (5;5.6)	<0.001

Supplemental Table S2. *Comparison of clinical manifestations amongst participants with and without hypertension*

	**Total (n=312)**	**Hypertension (n=158)**	**No Hypertension (n=154)**	**p-value**
Age, years	54 (42.5;60)	58 (51.2;63)	46.5 (36;57)	<0.001
BMI, kg/m^2	26.9 (23.4;31.1)	29 (24.7;33.2)	25.0 (22.7;28.4)	<0.001
Ferritin	82.9 (35.27;155.85)	100 (58.5;177)	61.8 (20.2;132)	<0.001
Fibrinogen	3.490 (2.995;4.04)	3.67 (3.08;4.18)	3.34 (2.9;3.9)	0.001
HGB	134 (126;143)	136 (128;144)	131 (124;142)	0.01
WBC	5.927 (4.85;6.665)	5.96 (5.06;6.98)	5.49 (4.76;6.46)	0.01
DD	0.23 (0.19;0.36)	0.27 (0.19;0.438)	0.195 (0.19;0.3)	<0.001
NT-proBNP	0.043 (0.021;0.082)	0.0534 (0.0232;0.104)	0.0375 (0.0185;0.0659)	0.001
CRP	0.19 (0.09;0.41)	0.242 (0.117;0.64)	0.14 (0.06;0.29)	<0.001
Neutrophils	3.105 (2.49;3.895)	3.31 (2.55;4.00)	2.92 (2.39;3.69)	0.008
Fasting glucose	92.68 (85.04;103.07)	95.8 (88.6;113)	89.1 (82.6;97.5)	<0.001
HbA1c	5.3 (5.075;5.7)	5.6 (5.2;6.2)	5.2 (5;5.49)	<0.001
